# Role of NEU3 Overexpression in the Prediction of Efficacy of EGFR-Targeted Therapies in Colon Cancer Cell Lines

**DOI:** 10.3390/ijms21228805

**Published:** 2020-11-20

**Authors:** Federica Bovio, Samantha Epistolio, Alessandra Mozzi, Eugenio Monti, Paola Fusi, Matilde Forcella, Milo Frattini

**Affiliations:** 1 Department of Biotechnology and Biosciences, University of Milano-Bicocca, Piazza della Scienza 2, 20126 Milano, Italy; f.bovio@campus.unimib.it; 2 Laboratory of Molecular Pathology, Institute of Pathology, Via in Selva 24, 6600 Locarno, Switzerland; d.epistolio@tin.it (S.E.); milo.frattini@eoc.ch (M.F.); 3 Scientific Institute, IRCCS E. MEDEA, Bioinformatics, Via Don Luigi Monza 20, 23842 Bosisio Parini (LC), Italy; alessandra.mozzi@lanostrafamiglia.it; 4 Department of Molecular and Translational Medicine (DMTM), University of Brescia, Viale Europa 11, 25123 Brescia, Italy; eugenio.monti@unibs.it

**Keywords:** cetuximab, colorectal cancer, cell lines, sialidase NEU3, EGFR pathway

## Abstract

The epidermal growth factor receptor (EGFR), through the MAP kinase and PI3K-Akt-mTOR axis, plays a pivotal role in colorectal cancer (CRC) pathogenesis. The membrane-associated NEU3 sialidase interacts with and desialylates EGFR by promoting its dimerization and downstream effectors’ activation. Among the targeted therapies against EGFR, the monoclonal antibody cetuximab is active only in a subgroup of patients not carrying mutations in the MAP kinase pathway. In order to better understand the EGFR-NEU3 interplay and the mechanisms of pharmacological resistance, we investigated the role of NEU3 deregulation in cetuximab-treated CRC cell lines transiently transfected with NEU3 using Western blot analysis. Our results indicate that NEU3 overexpression can enhance EGFR activation only if EGFR is overexpressed, indicating the existence of a threshold for NEU3-mediated EGFR activation. This enhancement mainly leads to the constitutive activation of the MAP kinase pathway. Consequently, we suggest that the evaluation of NEU3 expression cannot entirely substitute the evaluation of EGFR because EGFR-negative cases cannot be stimulated by NEU3. Furthermore, NEU3-mediated hyperactivation of EGFR is counterbalanced by the administration of cetuximab, hypothesizing that a combined treatment of NEU3- and EGFR-targeted therapies may represent a valid option for CRC patients, which must be investigated in the future.

## 1. Introduction

The epidermal growth factor receptor (EGFR) plays a pivotal role in colorectal carcinogenesis. Upon binding to several ligands, EGFR allows the activation of two main downstream pathways: (i) the MAP kinase (MAPK) pathway, mainly involved in cell proliferation, by the sequential activation of KRAS, BRAF, MEK, ERK proteins, and (ii) the PI3K-PTEN-AKT pathway, mainly involved in cell survival through the activation of mTOR [1,2].

Monoclonal antibodies (MoAbs) against EGFR, such as cetuximab and panitumumab, have been developed and are able, by binding to EGFR extracellular domain, to prevent its activation, demonstrating high efficacy in metastatic colorectal cancer (mCRC), but only in a subgroup of patients [2]. To date, it has been widely demonstrated that alterations (i.e., point mutations) hyperactivating EGFR downstream effectors (such as KRAS, NRAS and BRAF) represent the main mechanism of primary resistance to these drugs: indeed, nowadays, cetuximab and panitumumab can be administered only in patients showing a RAS-BRAF wild-type pattern [3]. However, a wide group of patients, although carrying a favorable genetic profile of RAS and BRAF genes, are still not able to benefit from EGFR-targeted therapies [4,5,6,7]. These discrepancies upon EGFR-targeted therapies demonstrate that our knowledge about the mechanisms of EGFR activation and blockade in mCRC is still insufficient. At odds with lung cancer, in fact, at the moment, the molecular characterization of EGFR status does not seem to play any clinical role. Alterations of EGFR gene copy number and expression of EGFR-specific ligands have been evaluated in recent years and associated with response to MoAbs against EGFR, but not unequivocally approved by the scientific community [8,9,10,11,12].

A new mechanism of EGFR activation has recently been proposed: the level of sialylation. Sialic acids belong to a large family of related acidic sugars and are involved in a variety of biological events [13]. Sialic acid levels determine the negative charge of the cell surface and, apart from the enzymes involved in the acidic sugar biosynthesis, sialyltransferases and sialidases play pivotal roles in the fine tuning of sialoconjugates [14]. Together with other groups [15,16,17,18,19,20], we demonstrated that NEU3, a plasma membrane-associated sialidase, interacts with EGFR and modifies the levels of sialylation, with both direct and indirect mechanisms [21,22]. In particular, a better understanding of the regulation of EGFR glycosylation may provide novel insights into cancer biology and suggest possible therapeutic strategies, especially in the field of drugs against EGFR.

Therefore, the aim of the present study is the investigation of the role of NEU3 expression levels in the prediction of the efficacy of EGFR-targeted therapies, in terms of variation of cell viability and of an extensive molecular characterization of the EGFR downstream pathway deregulation, after cell transfection.

## 2. Results

### 2.1. Mutational Status of Colorectal Cell Lines

In order to identify the cells that can hypothetically benefit from the administration of EGFR-targeted therapies, we initially evaluated the mutational status of EGFR downstream pathways of 13 commercially available colon cancer cell lines, as well as a normal intestinal mucosa cell line. The results are summarized in Table 1. As expected, the normal intestinal mucosa cell line, CCD 841, did not show any alteration in the EGFR downstream pathways effectors. Among the colon cancer cell lines, six (SW403, SW116, SW480, SW1463, MICOL29 and SW620) were characterized by a mutation in exon 2 of the *KRAS* gene and two (CO115 and HT-29) by the V600E mutation in the *BRAF* gene. On the contrary, the five remaining colon cancer cell lines, namely SW48, DIFI, MICOL24, CACO-2 and E705, showed no hyperactivating mutations in *KRAS*, *NRAS*, *BRAF* and *PIK3CA* genes, with the E705 cell line carrying a silent mutation in *PIK3CA* gene. However, the *EGFR* gene of the SW48 cell line is hyperactivated by the presence of a G719S substitution in exon 18.

Therefore, all the cell lines carrying either a *KRAS* or *BRAF* or *EGFR* hyperactivating point mutation were excluded from subsequent experiments.

### 2.2. Sensitivity to Cetuximab

The four colon cancer cell lines without any alteration in the EGFR pathway (i.e., E705, DIFI, MICOL24, CACO-2), as well as the normal mucosa cell line, were treated with cetuximab in order to evaluate the effect of this drug. With the aim of estimating the drug concentrations required to inhibit 50% of the viability (IC50), a toxicological MTT test was performed.

**Figure 1:**
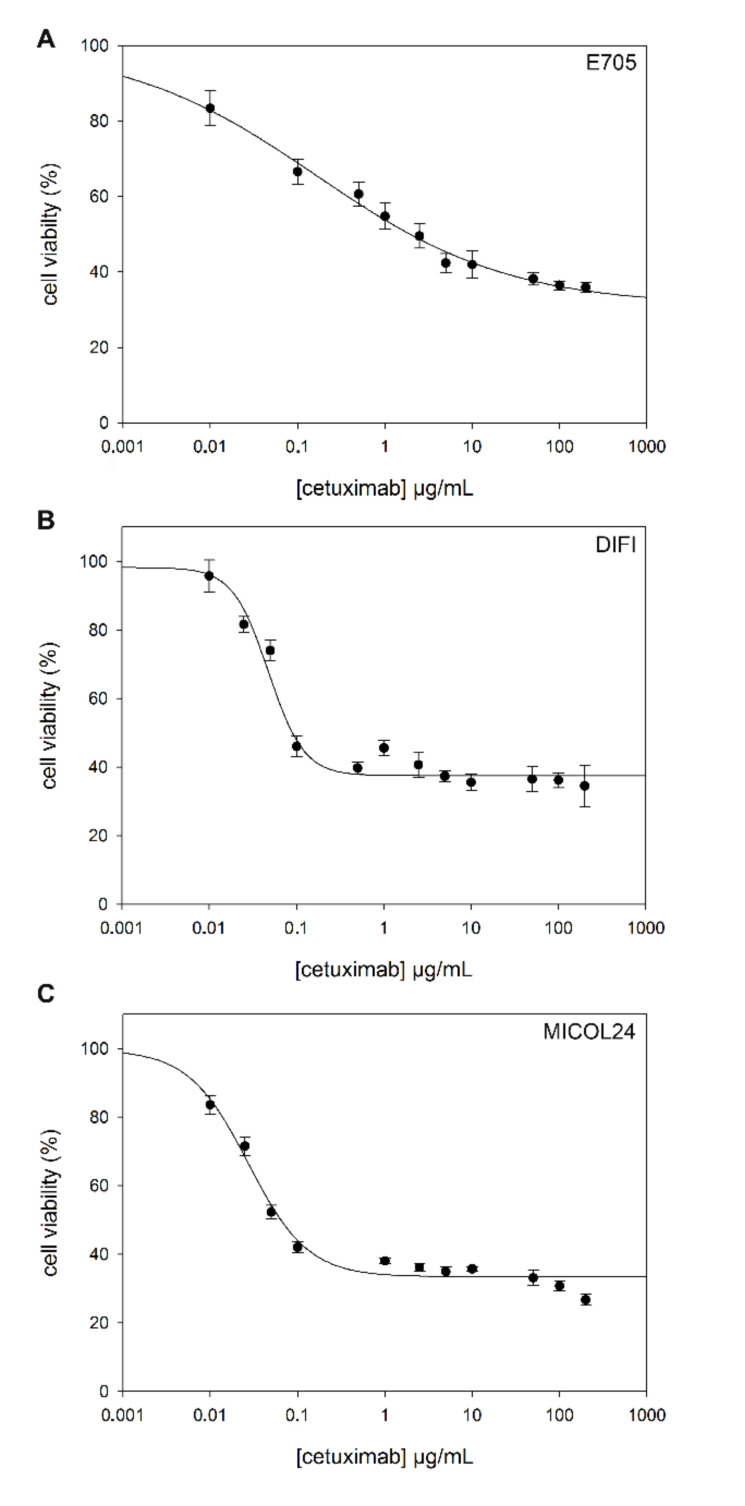
Evaluation of cetuximab IC50 by MTT test. Dose–response curves of human colon cell lines to cetuximab. Cell survival was determined after 36 h by MTT assay in the presence of different cetuximab doses (0–200 μg/mL). Nonlinear regression of experimental data for E705 (**A**), DIFI (**B**) and MICOL24 (**C**) cells lines was obtained using a four-parameter logistic curve f1 = min + (max − min)/(1 + (×/EC50)^(-Hillslope)). Data are shown as means ± standard error (SE).

Cells were seeded in 96-well plates and subjected to treatment with cetuximab at different concentrations for 36 h. Their viability was then evaluated. Three of the five cell lines, namely DIFI, MICOL24 and E705, showed higher sensitivity to cetuximab (Figure 1). The IC50 values for these cell lines are 0.048 ± 0.005 µg/mL for DIFI, 0.027 ± 0.004 µg/mL for MICOL24 and 0.165 ± 0.047 µg/mL for E705 (Table 2). On the contrary, the CACO-2 cell line, as well as the normal intestinal mucosa cell line CCD 841, did not show any significant variation in cell viability after treatment with cetuximab (IC50 > 200 µg/mL).

### 2.3. NEU3 Overexpression Effect on Cell Viability in the Presence or the Absence of Cetuximab

In order to investigate the impact of NEU3 overexpression on cell viability and upon cetuximab administration, all the cell lines were transfected with the following constructs: pcDNA3.1 (mock) or pcDNA3.1-HsNEU3, which lead to a transient NEU3 overexpression.

Four hours after transfection, the cell lines were treated with cetuximab at the concentration determined on the basis of viability assays. In particular, we used a concentration of cetuximab of 0.1 μg/mL for DIFI and MICOL24 cell lines, 1 μg/mL for E705 and 200 μg/mL for CCD 841 and CACO-2. Thirty-six hours after cetuximab administration, cell viability was determined using the toxicological MTT test and the effect of NEU3 in the presence or the absence of cetuximab was analyzed.

**Figure 2:**
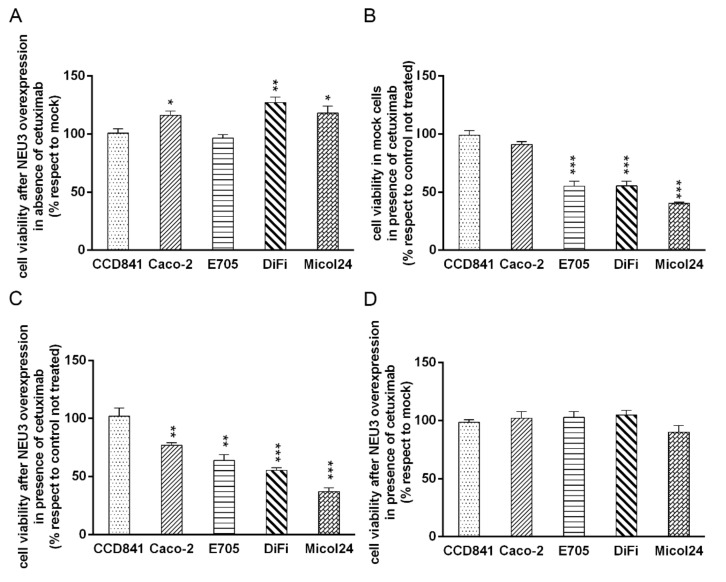
Evaluation of cell viability by MTT test after NEU3 overexpression and cetuximab administration. MTT tests were performed on CCD 841, CACO-2, E705, DIFI and MICOL24 cell lines transfected with either empty vector (mock) or pcDNA3.1-Hs NEU3 and treated or not with 200 μg/mL (CCD 841 and CACO-2), 1 μg/mL (E705) or 0.1 μg/mL (DIFI and MICOL24) of cetuximab for 36 h post-transfection. Cell viabilities after transfection in the presence or the absence of cetuximab (**A**,**C**) (data were normalized on control cells transfected with the empty vector). Cell viabilities of mock and NEU3 transfected cells after treatment with cetuximab (**B**,**D**) (data were normalized on cells without drug). Values are presented as means ± standard error (SE). * *p* < 0.05, ** *p* < 0.01, *** *p* < 0.001.

Upon transfection with the NEU3 construct, in the absence of cetuximab, three colon cancer cell lines, namely CACO-2, DIFI and MICOL24, showed an increase of cell viability, whereas the remaining colon cancer cell line E705, as well as the normal intestinal mucosa cell line CCD 841, did not show any variation (Figure 2A).

Upon transfection with empty pcDNA3.1 (mock), mimicking endogenous NEU3 expression, we observed that cetuximab caused a significant reduction in viability in E705, DIFI and MICOL24 cells (Figure 2B). When NEU3 was transiently overexpressed, we observed a 20% reduction in cell viability in the presence of cetuximab also in the CACO-2 cell line (Figure 2C). The healthy cell line showed no variation in cell viability in all the conditions investigated (Figure 2B,C).

After treatment with cetuximab, all colon cell lines showed no variation in cell viability upon NEU3 transfection, indicating that the increase in cell viability caused by NEU3 overexpression is counterbalanced by the effect of the drug. The normal mucosa cell line, previously unaffected by either NEU3 transfection or cetuximab treatment, showed no cell viability variation under the combination of treatment with cetuximab and NEU3 overexpression (Figure 2D).

### 2.4. Effect of NEU3 Overexpression and Cetuximab on EGFR, Akt and ERK Phosphorylation

We finally evaluated the effect of NEU3 overexpression, with or without cetuximab administration, on the phosphorylation of EGFR and its main downstream effectors, such as Akt and ERK.

**Figure 3:**
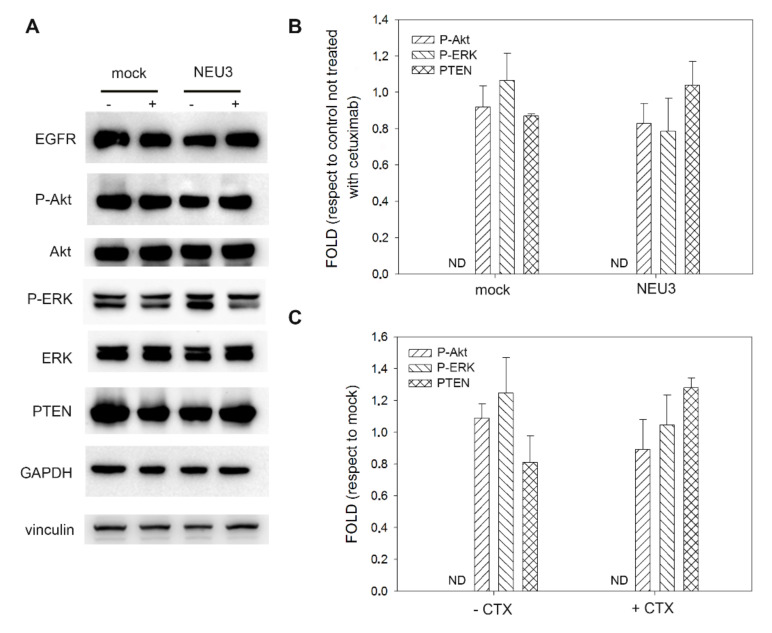
Western blot analysis of the CCD 841 cell line. (**A**) Representative Western blot analyses performed on the healthy mucosa colon cell line transfected either with the empty vector (mock) or pcDNA3.1-HsNEU3. Cells were treated for 3 h with 1 μg/mL cetuximab. Protein extracts were separated on a 12% SDS-PAGE and probed with anti-EGFR, anti-P-Akt, anti-Akt, anti P-ERK, anti-ERK and anti-PTEN antibodies. GAPDH and Vinculin were used as loading controls. The experiments were performed in triplicate. (**B**,**C**) Densitometric analysis was performed with Scion Image Software. Values are expressed by comparing the data obtained after cetuximab treatment with those obtained without cetuximab administration (**B**) by comparing the data obtained after transfection with NEU3 with those obtained after transfection with the empty vector (mock) (**C**). Values are presented as means ± standard error (SE).

As expected, in the normal intestinal mucosa CCD 841 cell line, neither treatment with cetuximab nor sialidase overexpression affected Akt and ERK phosphorylation levels. Moreover, P-EGFR was not detected, although the receptor is present in its unphosphorylated form (Figure 3A).

**Figure 4:**
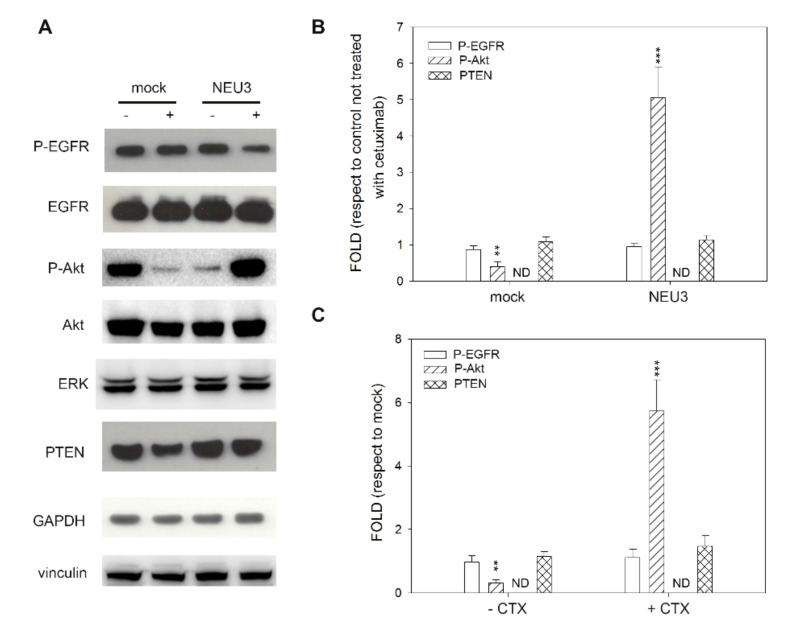
Western blot analysis of the CACO-2 cell line. (**A**) Representative Western blot analyses performed on the CACO-2 colon cancer cell line transfected either with the empty vector (mock) or pcDNA3.1-HsNEU3. Cells were treated for 3 h with 1 μg/mL cetuximab. Protein extracts were separated on a 12% SDS-PAGE and probed with anti-P-EGFR, anti-EGFR, anti-P-Akt, anti-Akt, anti-ERK and anti-PTEN antibodies. GAPDH and Vinculin were used as loading controls. The experiments were performed in triplicate. (**B**,**C**) Densitometric analysis was performed with Scion Image Software. Values are expressed by comparing the data obtained after cetuximab treatment with those obtained without cetuximab administration (**B**) by comparing the data obtained after transfection with NEU3 with those obtained after transfection with the empty vector (mock) (**C**). Values are presented as means ± standard error (SE). ** *p* <0.01, *** *p* < 0.001.

In colon cancer cell lines, three of them sensitive to cetuximab in the absence of NEU3 overexpression and all sensitive to the drug after NEU3 transfection, we observed great variability. CACO-2 cells did not show any variation in EGFR activation (P-EGFR), irrespective of the level of NEU3 expression and the administration of cetuximab. Regarding the downstream effectors of EGFR, P-ERK expression was always below the detection threshold. On the contrary, P-Akt showed a significant reduction after cetuximab administration in the absence of NEU3 expression, whereas a significant overexpression (fivefold) was observed after treatment with cetuximab in NEU3 transfected cells (Figure 4).

**Figure 5:**
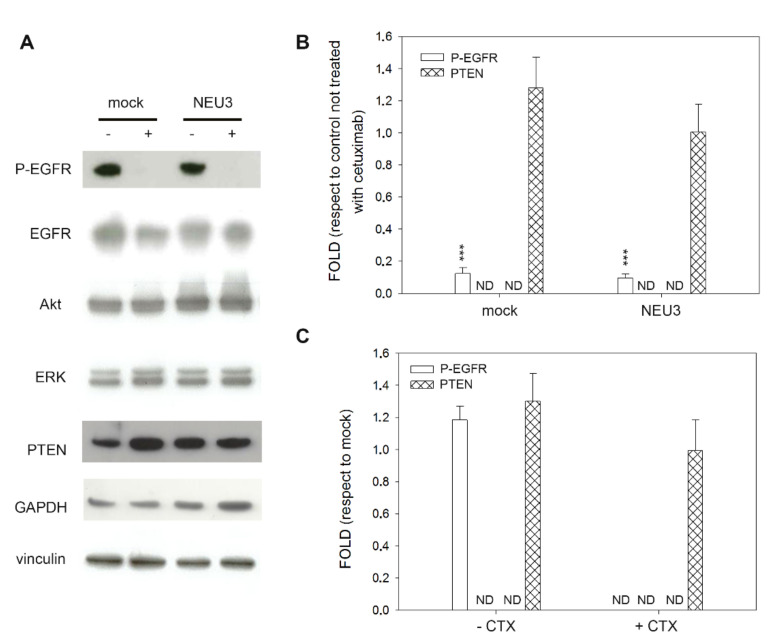
Western blot analysis of the E705 cell line. (**A**) Representative Western blot analyses performed on the E705 colon cancer cell line transfected either with the empty vector (mock) or pcDNA3.1-HsNEU3. Cells were treated for 3 h with 1 μg/mL cetuximab. Protein extracts were separated on a 12% SDS-PAGE and probed with anti-P-EGFR, anti-EGFR, anti-Akt, anti-ERK and anti-PTEN antibodies. GAPDH and Vinculin were used as a loading control. The experiments were performed in triplicate. (**B**,**C**) Densitometric analysis was performed with Scion Image Software. Values are expressed by comparing the data obtained after cetuximab treatment with those obtained without cetuximab administration (**B**); by comparing the data obtained after transfection with NEU3 with those obtained after transfection with the empty vector (mock) (**C**). Values are presented as means ± standard error (SE). *** *p* < 0.001.

The E705 cell line was characterized by a 90% decrease in P-EGFR levels after cetuximab treatment, independent of the NEU3 expression. Moreover, in the absence of cetuximab, NEU3 overexpression did not affect the EGFR phosphorylation level. On the contrary, the activation of EGFR downstream effectors Akt and ERK was always below the detection threshold, although the nonphosphorylated proteins were normally detected and did not show any variation (Figure 5).

The two remaining cell lines, DIFI and MICOL24, showed similar behaviors, with slight changes. In MICOL24 cells, the level of EGFR phosphorylation was substantially unaffected both after NEU3 transfection and after cetuximab administration, whereas in DIFI cells, a slight decrease was observed after cetuximab administration, with a fold decrease of 0.53 and 0.71 in mock and NEU3 overexpressing cells, respectively.

**Figure 6:**
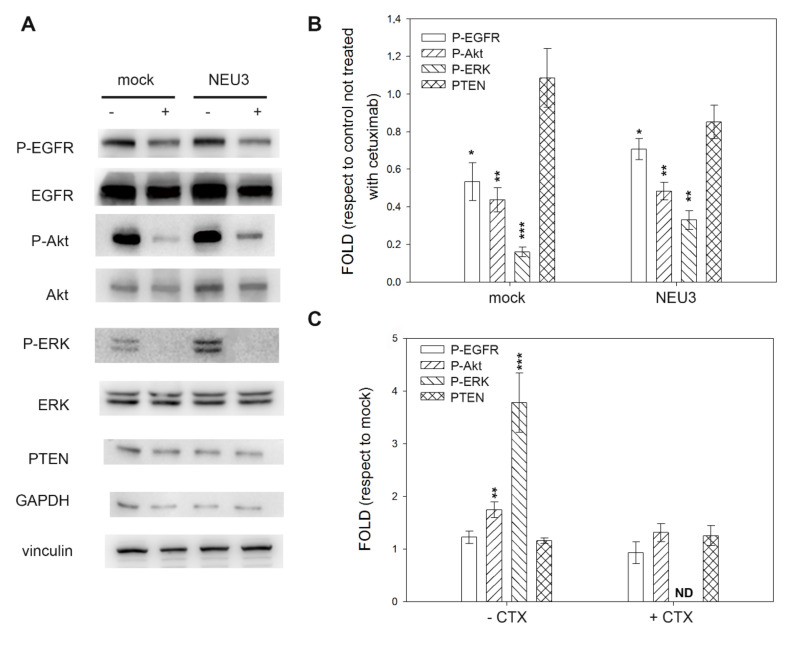
Western blot analysis of the DIFI cell line. (**A**) Representative Western blot analyses performed on the DIFI colon cancer cell line transfected either with the empty vector (mock) or pcDNA3.1-HsNEU3. Cells were treated for 3 h with 1 μg/mL cetuximab. Protein extracts were separated on a 12% SDS-PAGE and probed with anti-P-EGFR, anti-EGFR, anti P-Akt, anti-Akt, anti-P-ERK, anti-ERK and anti-PTEN antibodies. GAPDH and Vinculin were used as loading controls. The experiments were performed in triplicate. (**B**,**C**) Densitometric analysis was performed with Scion Image Software. Values are expressed by comparing the data obtained after cetuximab treatment with those obtained without cetuximab administration (**B**) by comparing the data obtained after transfection with NEU3 with those obtained after transfection with the empty vector (mock) (**C**). Values are presented as means ± standard error (SE). * *p* < 0.05, ** *p* < 0.01, *** *p* < 0.001.

**Figure 7:**
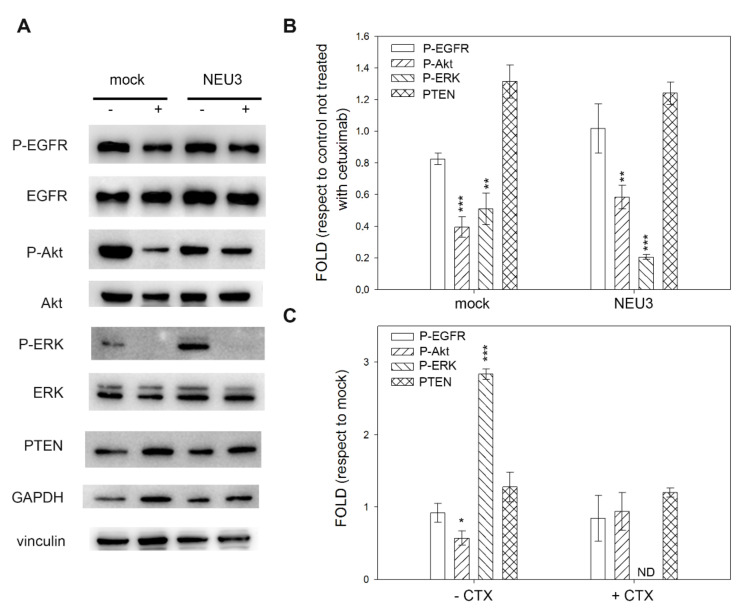
Western blot analysis of MICOL24 cell line. (**A**) Representative Western blot analyses performed on the MICOL24 colon cancer cell line transfected either with the empty vector (mock) or pcDNA3.1-HsNEU3. Cells were treated for 3 h with 1 μg/mL cetuximab. Protein extracts were separated on a 12% SDS-PAGE and probed with anti-P-EGFR, anti-EGFR, anti P-Akt, anti-Akt, anti-P-ERK, anti-ERK and anti-PTEN antibodies. GAPDH and Vinculin were used as loading controls. The experiments were performed in triplicate. (**B**,**C**) Densitometric analysis was performed with Scion Image Software. Values are expressed by comparing the data obtained after cetuximab treatment with those obtained without cetuximab administration (**B**) by comparing the data obtained after transfection with NEU3 with those obtained after transfection with the empty vector (mock) (**C**). Values are presented as means ± standard error (SE). * *p* < 0.05, ** *p* < 0.01, *** *p* < 0.001.

The activation of Akt was significantly diminished after cetuximab treatment, irrespective of NEU3 overexpression in both cells. A reduction in the ERK phosphorylation level was also observed in both cell lines, stronger in mock DIFI cells (fold decrease of 0.16 and 0.33 for mock and NEU3, respectively) and in the presence of NEU3 overexpression in MICOL24 cells (fold decrease of 0.51 and 0.20 for mock and NEU3, respectively). Moreover, we observed ERK activation after NEU3 overexpression in the absence of cetuximab, with a fold increase of 3.8 and 2.8 in DIFI and MICOL24 cells, respectively (Figure 6 and Figure 7).

In all the cell lines and upon every treatment, PTEN levels were substantially unaffected.

## 3. Discussion

The identification of patients affected by mCRC who can really benefit from the administration of MoAbs against EGFR (cetuximab and panitumumab) is a matter of debate. At odds with all the other targeted therapies, including tyrosine kinase inhibitor (TKI), which is able to block the same molecule (EGFR) in another cancer type (non-small-cell lung cancer), mCRC patients addressed to MoAbs against EGFR are selected “by subtraction.” Indeed, only patients characterized by the absence of mutations in one of the EGFR downstream pathways (the MAP kinase axis, investigated in *RAS* and *BRAF* genes) can be treated with these therapies [23]. Even if the elimination of patients with a downstream constitutive activation leading to insensitivity to anti-EGFR drugs with upstream activity is clearly understandable, it is currently impossible to identify the patients who can profit from the administration of these therapies in the absence of MAP kinase pathway activation. In fact, only 30% of *RAS*/*BRAF* wild-type patients experience a prolonged survival upon treatment with cetuximab/panitumumab [5,6,7]. This evidence comes from the inability to understand when EGFR is activated and, therefore, represents the driver of colorectal carcinogenesis. The activation of EGFR is, indeed, very complex. It has been reported that not only gene amplification but also a copy number gain may represent a molecular predictor of EGFR-targeted therapies’ efficacy [24]. However, in parallel, a lack of reproducibility among laboratories that used the same approach (fluorescent in situ hybridization) in a ring test [25] has been demonstrated, making *EGFR* gene status evaluation an unfeasible approach for the identification of patients who have constitutive activation of EGFR.

On the other hand, there is an increasing body of evidence that there are additional mechanisms of EGFR activation. The most recent involves the level of EGFR sialylation. In fact, in the vein of other international studies [15,16,17,18,19,20], we demonstrated that NEU3, a plasma-membrane-associated sialidase, is able to interact with EGFR leading to changes in EGFR sialylation that, in turn, with both direct and indirect mechanisms, are strictly linked to changes in EGFR activation [21]. The present study represents the natural extension of our previous analyses. Herein, we evaluated whether changes in EGFR sialylation may be associated with a modification of the sensitivity to EGFR-targeted therapies. As a starting point, we used different colon cancer cell models in order to mimic the interpersonal heterogeneity with respect to the administration of anti-EGFR MoAbs. In a panel of colon cancer cell lines available in our laboratory, we performed a complete mutational signature of EGFR downstream pathways and we selected four cell lines that are completely wild-type in *RAS*, *BRAF* and *PIK3CA* genes and that, therefore, are putatively sensitive to EGFR-targeted therapies. As a control, we included a normal intestinal mucosa cell line, characterized by a *RAS*/*BRAF*/*PIK3CA* wild-type status. These five cell lines were evaluated in terms of cetuximab sensitivity under normal conditions and after transfection with a plasmid containing NEU3 ORF and leading to transient enzyme overexpression. We finally evaluated the expression, as well as the activation, of EGFR, ERK, AKT and PTEN proteins through Western blot experiments under all the experimental conditions.

As expected, the normal intestinal mucosa cell line did not show any sensitivity to cetuximab. The four colon cancer cell lines experienced a different behavior with respect to the drug, mimicking the situation that is routinely observed in patients. In particular, DIFI, MICOL24 and E705 cell lines showed sensitivity, in contrast to the CACO-2 cell line that did not show any significant variation in cell viability after treatment with cetuximab. This different behavior among the cell lines cannot be explained only by the *EGFR* status. In fact, we previously published the characterization of *EGFR* gene status and gene expression in these cell lines [21]: DIFI and MICOL24 (cetuximab-sensitive) are characterized by *EGFR* gene amplification and gene overexpression, CACO-2 (cetuximab insensitive) shows a similar *EGFR* gene status with respect to MICOL24 and DIFI cell lines, whereas E705 (cetuximab-sensitive) is characterized by a low level of *EGFR* gene expression. On the other hand, the level of NEU3 expression cannot completely explain our data. In fact, if the E705 cell line is characterized by NEU3 overexpression that leads to EGFR activation [21] and, as a consequence, to cetuximab sensitivity, the CACO-2 cell line is also characterized by NEU3 overexpression. In other words, although the CACO-2 cell line seems to have all the positive features to be cetuximab-sensitive, namely EGFR overexpression and activation as well as NEU3 overexpression, its viability is completely unaffected after treatment with EGFR MoAbs. This indicates the presence of a different mechanism of primary resistance that can prevent the efficacy of cetuximab in a supposedly positive context in terms of deregulation at EGFR level.

When we forced NEU3 overexpression upon transfection with a plasmid containing the NEU3 transcript, we observed an increase in cell viability, with respect to the transfection with an empty vector (mock), only in cells characterized by high levels of EGFR expression, namely DIFI, MICOL24 and CACO-2. In fact, the cells characterized by low levels of EGFR, such as the E705 colon cancer cell line and the normal intestinal mucosa CCD 841 cell line, did not show any change in cell viability, suggesting that NEU3 is unable to hyperactivate EGFR if the receptor is present at low levels and already activated by the endogenous level of the enzyme. We therefore suggest that if EGFR is overexpressed, its activation can be increased by NEU3, but if EGFR is in a normal status, as in the normal intestinal mucosa, NEU3 activity is not able to transform EGFR into a cancer driver.

After NEU3 transfection and cetuximab administration, we observed a significant decrease in viability not only in cells naturally sensitive to the drug such as DIFI, MICOL24 and E705 but also in cells naturally insensitive to cetuximab like CACO-2, supporting the hypothesis that in the presence of a strong EGFR overexpression, the endogenous level of NEU3 is not able to saturate the interaction and therefore the activation of the receptor. Indeed, the surplus of activation we observed in CACO-2 cells transfected with NEU3 is abolished by the treatment with cetuximab, indicating that a NEU3-mediated hyperactivation of EGFR is counterbalanced by the administration of EGFR-targeted therapies.

We finally evaluated the biochemical changes in the activation of EGFR and its main downstream effectors, namely ERK for the MAP kinase pathway, Akt and PTEN for the mTOR pathway, after NEU3 overexpression, and in the presence or the absence of cetuximab administration. No changes were observed in the normal intestinal mucosa cell line, confirming that in the presence of low basal EGFR expression, NEU3 does not contribute to the overstimulation of this pathway.

The situation of colon cancer cell lines is complex, and in all four cases, we observed different aspects. Two cell lines, DIFI and MICOL24, were the most similar, showing the MAP kinase pathway as the most important pathway. NEU3 overexpression stimulated ERK phosphorylation, and this activation was completely abolished after cetuximab treatment. On the contrary, weak activation of Akt was detected in DIFI cells upon NEU3 transfection, which was abolished by cetuximab regardless of the level of NEU3 expression. No Akt stimulation was observed in the MICOL24 cell line. The third sensitive colon cancer cell line, E705, showed that EGFR phosphorylation was not affected by NEU3 transfection, and cetuximab acted on cell viability regardless of NEU3 transfection. Interestingly, at odds with the nonphosphorylated proteins that were correctly detected and did not show any changes under the different treatments, the active forms of ERK and AKT could not be detected by Western blot experiments. The CACO-2 cell line did not show any variation in EGFR activation (P-EGFR), irrespective of the level of NEU3 expression and cetuximab treatment, indicating that other, unknown, mechanisms of EGFR activation are present. The ERK phosphorylated form was not detected, whereas an abnormal overexpression of the phosphorylated form of AKT was observed after cetuximab administration in NEU3 transfected cells. The biochemical results of E705 and CACO-2 cell lines mirror the observations done in terms of cell viability.

Our data reinforce the correlation between EGFR and NEU3, strengthening the hypothesis that NEU3 may play a role in cell migration, probably by acting on EGFR. The precise mechanism has not yet been clarified. In fact, a recent contribution demonstrated that the inhibition of NEU3 activity caused significant retardation of cell migration in breast cancer and prostate cancer cell lines [26], and another study revealed that NEU3 forced overexpression acts on Crumbs3 in modulating cell migration of colon cancer cells [27]. However, if NEU3 acts directly or through EGFR, it needs more studies.

Our data cannot be explained by different levels of EGFR sialylation. In our previous study [21], we demonstrated that the level of EGFR α2,6-sialylation was reduced in cells overexpressing the active form of the NEU3 sialidase. This result was obtained using a lectin-binding assay based on biotinylated *Sambucus nigra*. No reduction in EGFR sialylation was detected following transfection with the inactive double-mutant form of NEU3. Furthermore, these data were confirmed by analysis in MALDI-TOF mass spectrometry, which strongly suggests that EGFR sialylation is regulated by NEU3. Park and colleagues indicated that β-galactoside α2-6-sialyltransferase (ST6Gal-I) affects EGFR activation by altering its sialylation level. In particular, ST6Gal-I knockdown enhanced EGFR phosphorylation, promoting a more rapid ERK activation and affecting the efficacy of anti-EGFR therapeutic agents [28].

On the other hand, the controversial results we observed in terms of the activation of EGFR downstream pathways cannot be ascribed to the influence of tumor location. In fact, it has been proposed that the left-sided colon may profit more from EGFR-targeted therapies, although characterized by lower levels of EGFR expression [29]. However, this preliminary datum has not been sustained by a meta-analysis of clinical trials [30,31]. Moreover, there is a lack of information about NEU3 expression and tumor sidedness in colorectal carcinogenesis [32].

## 4. Materials and Methods

### 4.1. Cell Cultures

E705 and MICOL24 colon cancer cells (kindly provided by Fondazione IRCCS Istituto Nazionale dei Tumori, Milan, Italy) were grown in RPMI 1640 medium supplemented with heat-inactivated 10% fetal bovine serum (FBS), 2 mM l-glutamine, 100 U/mL penicillin, 100 μg/mL streptomycin and were maintained at 37 °C in a humidified 5% CO_2_ incubator. The CACO-2 (ATCC^®^ HTB-37™) colon cancer cell line and CCD 841 (ATCC^®^ CRL-1790™) healthy mucosa cell line were grown in EMEM medium supplemented with heat-inactivated 10% FBS, 2 mM l-glutamine, 1% nonessential amino acids, 100 U/mL penicillin, 100 μg/mL streptomycin and maintained at 37 °C in a humidified 5% CO_2_ incubator. DIFI human CRC cells, kindly provided by Josep Tabernero (Vall d’Hebron Institute of Oncology, Vall d’Hebron University Hospital, Universitat Autònoma de Barcelona, Spain), were grown in Ham’s F12 medium supplemented with heat-inactivated 5% FBS, 2 mM l-glutamine, 100 U/mL penicillin, 100 μg/mL streptomycin and were maintained at 37 °C in a humidified 5% CO_2_ incubator. ATCC^®^ validated cell lines by short tandem repeat profiles that are generated by simultaneous amplification of multiple short tandem repeat loci and amelogenin (for gender identification). All the reagents for cell culture were supplied by Lonza (Lonza Group, Basel, Switzerland).

### 4.2. Mutational Status of EGFR Pathway

Genomic DNA from each cell line was isolated using the QIAamp Mini Kit (Qiagen, Chatsworth, CA, USA). The extracted DNA was quantified by Nanodrop and was diluted to 25 ng/μL. After the dilution, we investigated point mutations in the most relevant genes of the EGFR pathway: *KRAS* (exons 2-3-4), *NRAS* (exons 2-3-4), *BRAF* (exons 11 and 15) and *PIK3CA* (exons 9 and 20). The direct sequencing of PCR products was based on the Sanger sequencing method and was done using a 3130 Genetic Analyzer (Applied Biosystems, Foster City, CA, USA) according to the protocols previously published [24,33,34,35]. The resulting electropherograms were analyzed with the appropriate software (SeqScape Software Version 2.5TM, Applied Biosystems, Foster City, CA, USA). Each sequence reaction was performed at least twice, starting from independent PCR reactions in order to confirm the DNA sequence.

### 4.3. Vector

The cDNA coding for human sialidase NEU3 was previously subcloned into plasmid pcDNA3.1 (Invitrogen, Carlsbad, CA, USA), in frame with C-terminal haemagglutinin epitope [36].

### 4.4. Transfection

Cells were seeded at 6 × 10^5^ cells/60 mm dish or at 1 × 10^4^ cells/well into 96-well plates and, after 24 h, transiently transfected with pcDNA3.1 vector containing wild-type NEU3 cDNA or with the empty vector as a control (mock) in complete medium using JetPEI^TM^ DNA transfection reagent (Polyplus Transfection SA, Illkirch, France), according to the manufacturer’s instructions.

### 4.5. Viability Assay

Cell viability was investigated using in vitro toxicology assay kit MTT based (Sigma, St. Louis, MO, USA), according to the manufacturer’s protocols.

In order to evaluate cetuximab sensitivity, cells were seeded in 96-well microtiter plates at a density of 1 × 10^4^ cells/well and, after 24 h, were treated with various cetuximab concentrations (0–200 μg/mL). After 36 h at 37 °C, the medium was replaced with a complete medium without phenol red, and 10 μL of 5 mg/mL MTT (3-(4,5-dimethylthiazol-2)-2,5-diphenyltetrazolium bromide) solution (Sigma, St. Louis, MO, USA) was added to each well. After a further 4 h incubation time, absorbance upon formed formazan crystals solubilization with 10% Triton-X-100 in acidic isopropanol (0.1 N HCl) was measured at 570 nm using a microplate reader. Viabilities were expressed as a percentage of the untreated controls. The 50% growth inhibition (IC50) was determined from the dose–response curve. Each experiment was performed in three replicate wells for each drug concentration, and the results are presented as the mean of at least three independent experiments.

In order to evaluate the effect of NEU3 on cell viability in the presence of 0.1 μg/mL cetuximab for DIFI and MICOL24, 1 μg/mL for E705 and 200 μg/mL for CCD 841 and CACO-2, cells were seeded in 96-well plates at a density of 1 × 10^4^ cells/well and after 24 h were transiently transfected. After 4 h from the addition of the jetPEI/DNA mixture to the cells, the medium was changed and the cells were treated with the corresponding concentration of cetuximab. After incubation at 37 °C for 36 h post-transient transfection, the medium was replaced with complete medium without phenol red, and 10 μL of 5 mg/mL MTT solution was added to each well. After a further 4 h incubation time, absorbance upon solubilization was measured at 570 nm using a microplate reader. Viabilities were expressed as a percentage of the mock.

Cetuximab (Erbitux^®^) was purchased from Merck (Merck, Darmstadt, Germany).

### 4.6. SDS-PAGE and Western Blot

To examine the effect of sialidase NEU3 and cetuximab on the phosphorylation of EGFR and EGFR downstream members, the human colon cancer cell lines E705, MICOL24, CACO-2 and DIFI and the normal human colon cell line CCD 841 were seeded at 6 × 10^5^ cells/60 mm dish, transiently transfected and 36 h after transfection treated with 1 μg/mL cetuximab for 3 h.

The cells were then rinsed with ice-cold PBS and lysed in RIPA buffer containing 1 μM leupeptin, 2 μg/mL aprotinin, 1 μg/mL pepstatin, 1 mM PMSF (phenylmethylsulfonyl fluoride) and phosphatase inhibitors. After lysis on ice, homogenates were obtained by passing 5 times through a blunt 20-gauge needle fitted to a syringe and centrifuged at 14,000× *g* for 30 min. Supernatants were analyzed for protein content by the BCA protein assay [37].

SDS-PAGE and Western blot were performed by standard procedures [38]. Twenty or sixty micrograms of proteins were separated on 12% acrylamide/bis-acrylamide SDS-PAGE and transferred onto a nitrocellulose membrane (Millipore, Billerica, MA, USA). The membrane was subsequently blocked for 30 min in 5% (*w*/*v*) dried milk in PBS and incubated overnight at 4 °C probed with the appropriate primary antibodies. The following primary antibodies (all purchased by Cell Signaling Technology, Danvers, MA, USA) were used: anti-EGFR (dilution 1:1000), phospho-EGFR (Tyr1068; dilution 1:1000), p44/42 MAPK (ERK 1/2; dilution 1:1000), phospho-p44/42 MAPK (ERK 1/2) (Thr202/Tyr204; dilution 1:1000), Akt (dilution 1:1000), phospho-Akt (Ser473; dilution 1:1000), PTEN (dilution 1:1000), vinculin (dilution 1:10,000) and GAPDH (dilution 1:10,000). After three washing (10 min each) in PBS, 0.3% (*v*/*v*) Tween 20, the membrane was incubated for 1 h with IgG HRP-conjugated secondary antibodies diluted 1:10,000 (purchased by Cell Signaling Technology, Danvers, MA, USA). After three washing (10 min each) in PBS, 0.3% (*v*/*v*) Tween 20, proteins were visualized using ECL detection system (EuroClone, Pero, Milan, Italy), according to the manufacturer’s instructions. Protein levels were quantified by densitometry of immunoblots using Scion Image software (Scion Corp., Frederick, MD, USA).

### 4.7. Statistical Analysis

All the experiments were performed in biological triplicates, and the data were analyzed using a two-way ANOVA followed by a Tukey post hoc test. Results were considered statistically significant at *p* < 0.05.

## 5. Conclusions

Our results help to clarify the interplay between EGFR and NEU3. In the absence of EGFR expression, or when the receptor is expressed at a very low level, as in normal cells, NEU3 cannot transform EGFR into a cancer driver, whereas NEU3 can help the activation of EGFR if the receptor is overexpressed, indicating the existence of a threshold for NEU3-mediated EGFR activation. Consequently, we suggest that the evaluation of NEU3 expression cannot entirely substitute the evaluation of EGFR because EGFR-negative cases cannot be stimulated by NEU3. As regards to the downstream pathways, we observed great variability, but our data indicate that the main axis is the MAP kinase pathway. This is conceivable because the mutations occurring in the three main members of this pathway, namely *KRAS*, *NRAS* and *BRAF*, are the only changes that are useful at the diagnostic level. More controversial, as also reported in the literature [39,40,41], is the deregulation of the PI3K/AKT/mTOR pathway, whose role needs to be studied in depth with ad hoc experiments. Overall, our results indicate that compounds targeting NEU3 should be studied in combination with EGFR-targeted therapies and not alone, as a single-agent therapy, because of the heterogeneity of mCRC and the complexity of the biochemical pathways involved. The inhibition of NEU3 activity is, however, far from full success. Despite the efforts of many research groups, only few NEU3 inhibitors have been developed and tested. More in detail, there is only one molecule (“Compound 4”) that selectively inhibits NEU3. Zanamivir, an antiviral and bacterial NEU inhibitor, has low micromolar activity against NEU3 but also against NEU2. 2,3-dehydro-2-deoxy-*N*-acetylneuraminic acid (DANA) is a pan-selective inhibitor of all human NEU isoenzymes with a modest preference for NEU3 and NEU4 [26]. Moreover, it has been demonstrated that the modulation of sialidase expression might be effectively achieved by the appropriate use of recombinant sialidases for upregulation or specific inhibitors, antibodies and siRNAs for downregulation [42]. In conclusion, only Compound 4 seems to be NEU3-specific and can be proposed for evaluation in combination with EGFR-targeted therapies. In this context, the use of tumor-derived xenografts may represent the best procedure for the preclinical evaluation of the combination of these drugs because more adherent to the real situation with respect to cell line models.

## Figures and Tables

**Table 1:** *KRAS*, *NRAS*, *BRAF*, *PIK3CA* and *EGFR* mutational status of colon cancer and normal intestinal mucosa cell lines.

Cell Line	*KRAS*	*NRAS*	*BRAF*	*PIK3CA*	*EGFR*
SW48	WT	WT	WT	WT	G719S
CO115	WT	WT	V600E	WT	WT
SW403	G12V	WT	WT	WT	WT
SW1116	G12A	WT	WT	WT	WT
SW480	G12V	WT	WT	WT	WT
SW1463	G12C	WT	WT	WT	WT
E705	WT	WT	WT	H1047H	WT
MICOL29	G12D	WT	WT	WT	WT
DIFI	WT	WT	WT	WT	WT
MICOL24	WT	WT	WT	WT	WT
HT-29	WT	WT	V600E	WT	WT
SW620	G12V	WT	WT	WT	WT
CACO-2	WT	WT	WT	WT	WT
CCD 841	WT	WT	WT	WT	WT

**Table 2:** Cetuximab IC50 values determined in all cell lines.

Cell Lines	IC50 (µg/mL, Mean ± SEM)
CCD 841	>200
CACO-2	>200
E705	0.165 ± 0.047
DIFI	0.048 ± 0.005
MICOL24	0.027 ± 0.004

Note: IC50 was defined as the concentration that resulted in a 50% decrease of viability in MTT assay.
